# Supplementary material for: Shifting patterns of seasonal influenza epidemics
Source: Sci Rep. 2018 Aug 24;8:12786. doi: 10.1038/s41598-018-30949-x (PMC6109160; doi:10.1038/s41598-018-30949-x)
Supplement: Supplementary file 1 — Supplementary information [file 41598_2018_30949_MOESM1_ESM.pdf]

Supporting information for: Shifting patterns of seasonal influenza epidemics

Pietro Coletti[1,*], Chiara Poletto[2], Clément Turbelin[2], Thierry Blanchon[2], and Vittoria Colizza[2]

[1]ISI Foundation, Turin, Italy
[2]INSERM, Sorbonne Université, Institut Pierre Louis dEpidémiologie et de Santé Publique IPLESP, F75012 Paris, France
[*]Current address Universiteit Hasselt, I-Biostat, 3500 Hasselt, Belgium

1 Supplementary Tables

Table S1: Summary of influenza seasons. We classify excess attack rate according to the distribution and its percentiles; weak, medium, and strong attack rates indicate values within the 33th percentile, between the 33th and the 66th, and above the 66th, respectively.

	Peak time category	Time from onset to peak (weeks)	Attack rates for 100k population	Dominant subtype	Proxy of vaccine coverage
84-85	Winter	3	High	NA	NA
85-86	Late	7	High	H3	NA
86-87	Winter	3	Low	H1	NA
87-88	Late	3	Low	B	NA
88-89	Early	4	High	H1	NA
89-90	Early	4	High	H3	NA
90-91	Late	3	Low	B	NA
91-92	Early	2	Medium	H3	NA
92-93	Winter	4	Low	B	NA
93-94	Early	3	High	H3	NA
94-95	Late	3	Low	B	NA
95-96	Early	5	High	H1	NA
96-97	Early	4	High	H3	NA
97-98	Late	8	Medium	H3	NA
98-99	Late	7	High	H3	NA
99-00	Early	4	High	H3	NA
00-01	Winter	3	Low	H1	NA
01-02	Winter	3	Medium	H3	NA
02-03	Late	3	Low	B	NA
03-04	Early	4	Medium	H3	NA
04-05	Winter	4	High	H3	NA
05-06	Winter	2	Low	B	NA
06-07	Winter	4	Medium	H3	NA
07-08	Winter	5	Medium	H1	NA
08-09	Winter	5	Medium	H3	58,7
09-10	Early	6	Medium	H1	60,2
10-11	Early	3	Medium	B	51,8
11-12	Late	3	Low	H3	51,7
12-13	Winter	6	Medium	B	50,1
13-14	Late	2	Low	H1	48,9

Table S2: Benjamini-Hochberg reference values for Mantel test in Table 1 of the main paper. False discovery rate is corrected for at the $\alpha = 0.05$ level.

**All seasons**	p-val	Benjamini-Hochberg reference value	Significative
Distance	0.001	0.00125	YES
Commuters	0.019	0.025	YES
Flight passengers	0.78	0.0375	NO
Product of population	0.92	0.05	NO

**Group - clusters**	p-val	Benjamini-Hochberg reference value	Significative
Distance	0.001	0.00125	YES
Commuters	0.015	0.025	YES
Flight passengers	0.83	0.0375	NO
Product of population	0.97	0.05	NO

**Monoids**	p-val	Benjamini-Hochberg reference value	Significative
Distance	0.001	0.00125	YES
Commuters	0.66	0.025	NO
Flight passengers	0.85	0.0375	NO
Product of population	0.95	0.05	NO

2 Supplementary Figures

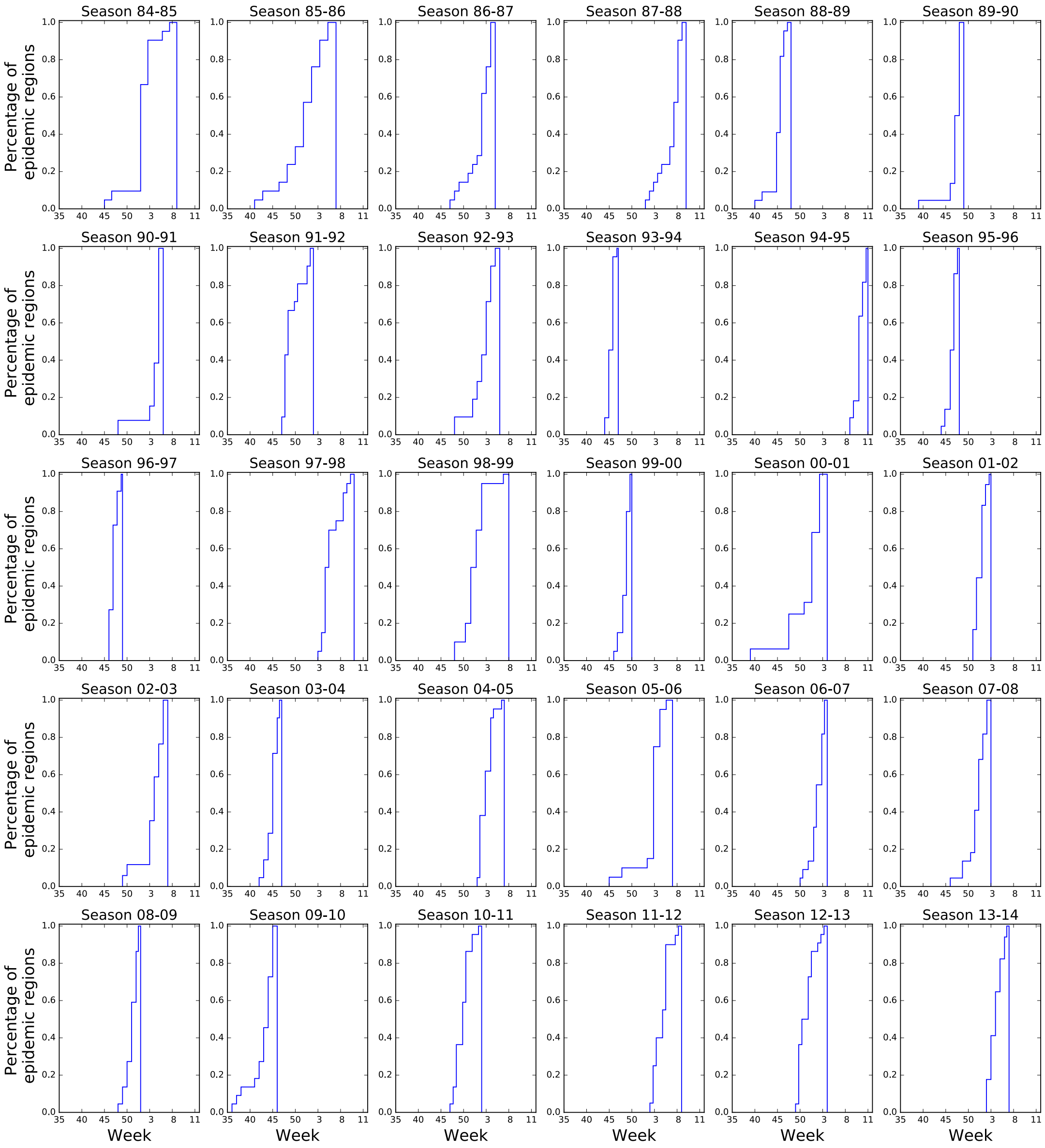

Figure S1. Diffusion of epidemic. In each plot we show the proportion of France regions above epidemic threshold over time. The x-axis has the same range for an easier comparison.

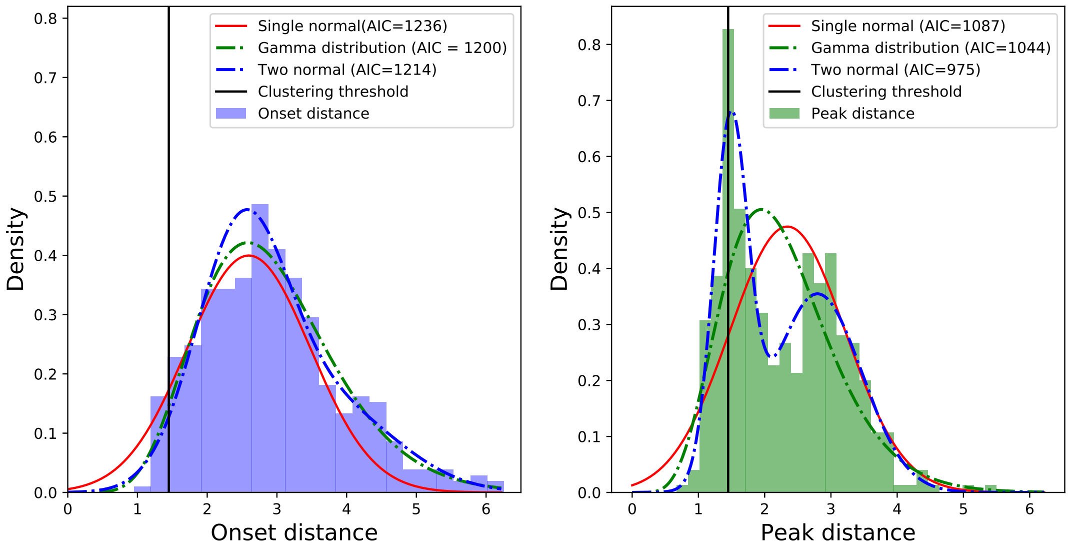

Figure S2. Onset and peak distances. Onset distance (left) and peak distance (right) distributions. The plot shows several models fit, with the corresponding AIC. The lowest AIC model is the Gamma for onset distance and a mixture of two normal distribution for the peak distance.

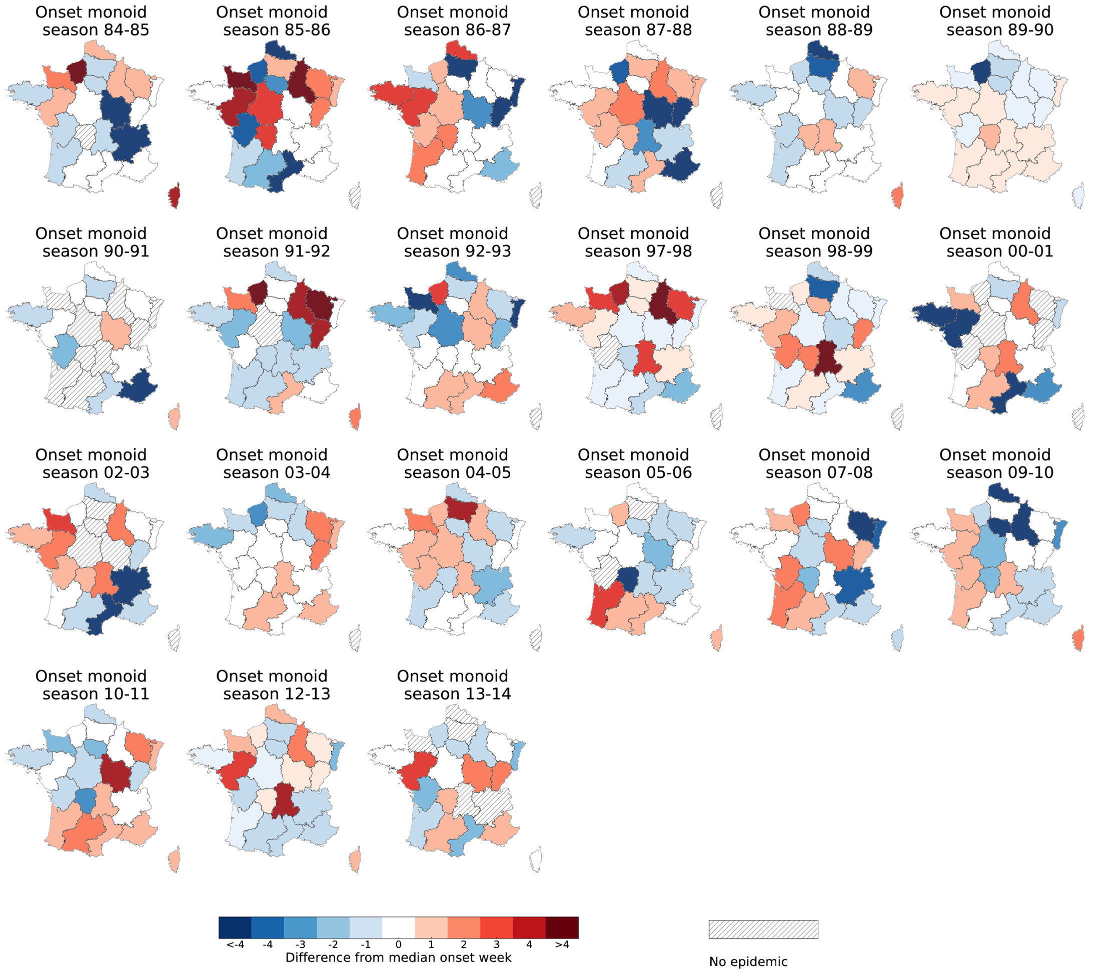

Figure S3. Geographic signature of onset monoids. Map of France showing the de-trended timing of onset monoids. White regions have a onset time that equals the median value over all the regions. Red regions have an onset later than the median and blu regions before. Regions having no epidemic are marked.

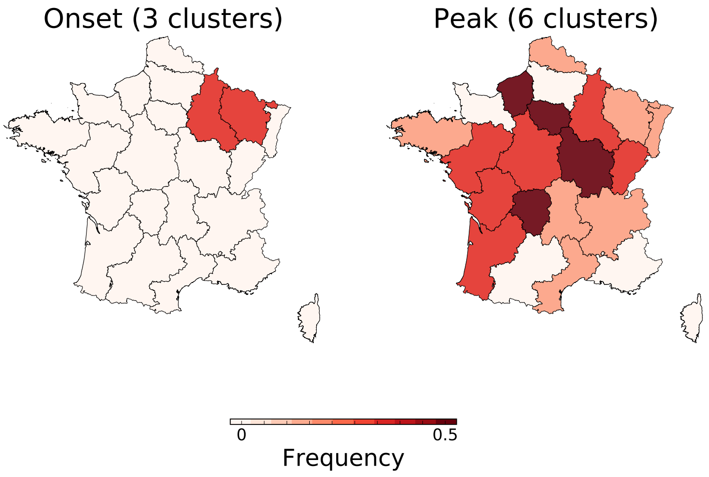

Figure S4. Frequency of having the same detrended timing for onset and peak clusters. France regions are colored according to the frequency with which they have the same detrended timing in onset clusters (left) and peak clusters (right).

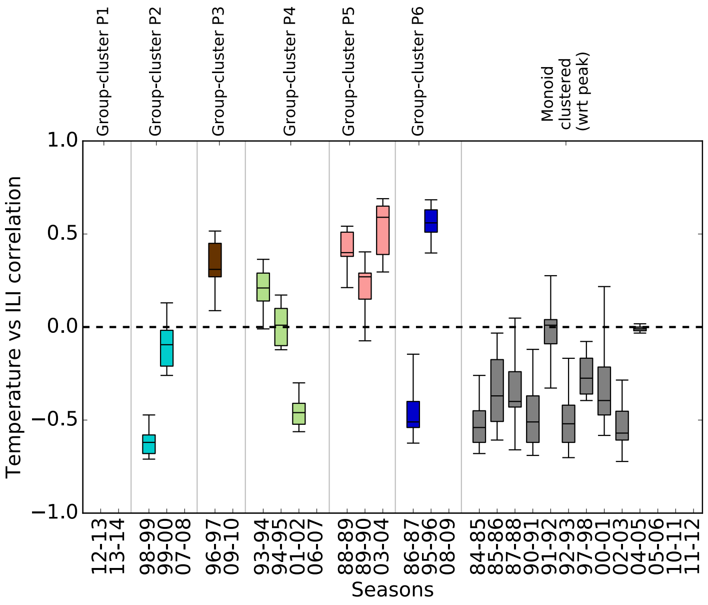

Figure S5. Box plot of correlation of regional ILI time series with regional temperature time series. Seasons that form group-clusters with respect to peak time are highlighted with the corresponding cluster color, while seasons that are monoid-clustered are shown in grey. Missing values are due to the temperature dataset, spanning seasons up to 2005 [1].

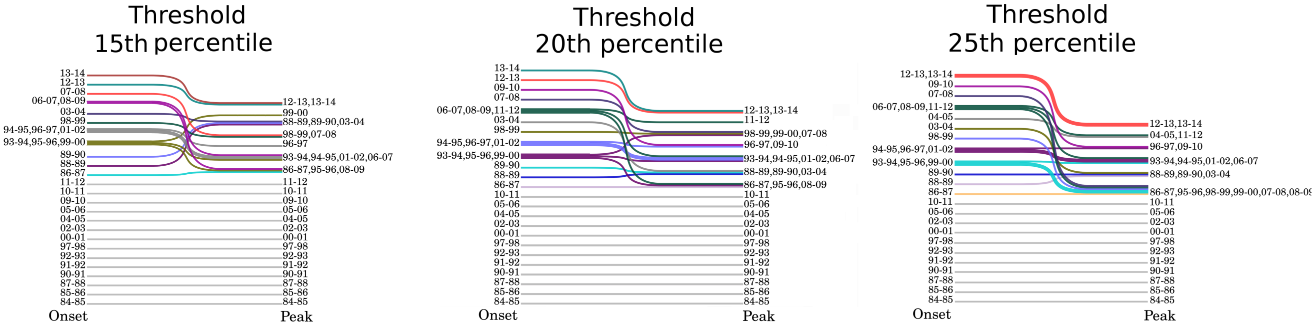

Figure S6. Alluvial diagrams for different clustering thresholds. Alluvial diagrams for three different values of the clustering threshold, respectively the 15th, 20th, and 25th percentile of the peak distance distribution. Variation of the clustering threshold has minor impact on results (only group clusters P6 and P3 merge for the largest value of the threshold).

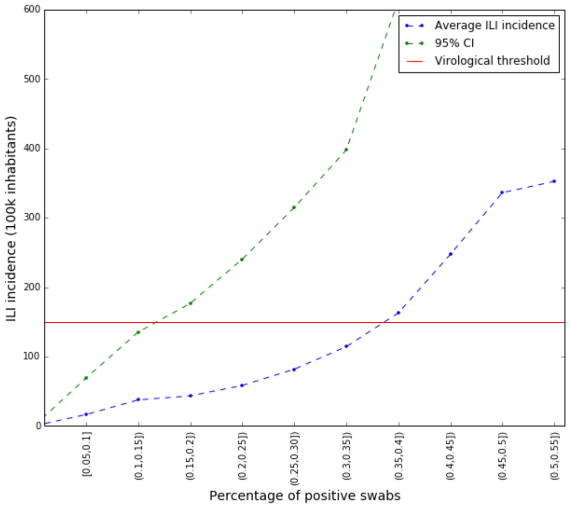

Figure S7. ILI incidence and laboratory confirmed cases. Percentage of positive swabs (binned) vs ILI incidence in the corresponding week. The horizontal line is the virological threshold of 150 cases for 100k inhabitants, lower than the 95% CI of the incidence in weeks with more than 15% of positive swabs.

References

[1] A. M. G. Klein Tank, J. B. Wijngaard, G. P. Knnen, R. Bhm, G. Demare, A. Gocheva, M. Mileta, S. Pashiardis, L. Hejkrlik, C. Kern-Hansen, R. Heino, P. Bessemoulin, G. Mller-Westermeier, M. Tzanakou, S. Szalai, T. Plsdttir, D. Fitzgerald, S. Rubin, M. Capaldo, M. Maugeri, A. Leitass, A. Bukantis, R. Aberfeld, A. F. V. van Engelen, E. Forland, M. Mietus, F. Coelho, C. Mares, V. Razuvaev, E. Nieplova, T. Cegnar, J. Antonio Lpez, B. Dahlstrm, A. Moberg, W. Kirchhofer, A. Ceylan, O. Pachaliuk, L. V. Alexander, and P. Petrovic. Daily dataset of 20th-century surface air temperature and precipitation series for the european climate assessment. *International Journal of Climatology*, 22(12):1441–1453, 2002.
